# Parallel Toxicities: A Comparative Analysis of Chemotherapy-Induced Neutropenia and Alopecia

**DOI:** 10.3390/cancers17071163

**Published:** 2025-03-30

**Authors:** Simonetta I. Gaumond, Karen J. Lee, Peyton V. Warp, Isabella Kamholtz, Emilee M. Dreifus, Joaquin J. Jimenez

**Affiliations:** 1 Department of Biochemistry and Molecular Biology, University of Miami Miller School of Medicine, Miami, FL 33136, USA; 2 Dr. Phillip Frost Department of Dermatology and Cutaneous Surgery, University of Miami Miller School of Medicine, Miami, FL 33136, USA; karen.lee@med.miami.edu (K.J.L.); pvw7@med.miami.edu (P.V.W.); irk122@miami.edu (I.K.); emileedreifus@med.miami.edu (E.M.D.)

**Keywords:** cancer, chemotherapy, neutropenia, febrile neutropenia, alopecia, hair loss

## Abstract

Chemotherapy-induced neutropenia (CIN) and chemotherapy-induced alopecia (CIA) are significant toxicities affecting cancer patients. CIN is a potentially fatal complication of chemotherapy caused by myelosuppression and increased infection susceptibility, while CIA, although not fatal, severely affects treatment adherence and mental health. This study provides a comprehensive comparative analysis of CIN and CIA, focusing on patient, disease, treatment, and genetic risk factors. Key risk factors for CIN and CIA include age, poor performance status, body mass index (BMI), laboratory abnormalities, and pre-existing comorbidities. Both toxicities were significantly associated with breast cancer patients, although CIN patients were more likely to have hematological cancer, and CIA patients were more likely to have solid tumors. Notably, anthracyclines, alkylators, and taxanes frequently induce both toxicities, although their timelines and clinical implications differed. There was no clear overlap between genetic predispositions and toxicities beyond single-nucleotide polymorphisms (SNPs) in the ABCB1 gene. This is the first study to directly compare CIN and CIA, offering insights into personalized oncology care. Understanding the risk factors implicated in the development of CIN and CIA will enable physicians to manage patient outcomes.

## Simple Summary

Chemotherapy-induced neutropenia (CIN) and chemotherapy-induced alopecia (CIA) are common side effects that can significantly impact cancer patients’ health and quality of life. CIN, a reduction in white blood cell counts, increases infection risks and can be life-threatening, while CIA, though not physically harmful, often affects patients’ mental well-being and treatment adherence due to the distress caused by hair loss. This review compares these two side effects, examining shared and unique risk factors related to patients, their conditions, and treatments. The findings aim to enhance understanding of these toxicities, support better risk assessment, and improve management strategies. This study has the potential to guide personalized cancer care, reduce treatment complications, and ultimately improve patient outcomes.

## 1. Introduction

Chemotherapy remains a cornerstone of cancer therapy, offering significant therapeutic benefits but often undermined by severe toxicities and side effects. Among these, chemotherapy-induced neutropenia (CIN) and chemotherapy-induced alopecia (CIA) are particularly distressing for both clinicians and patients. CIN is characterized by myelosuppression, which elevates the risk of infection, hospitalization, and mortality rates, severely complicating patient management [1,2]. In contrast, CIA, although not life-threatening, profoundly impacts patients’ quality of life, causing psychological distress that can hinder treatment adherence and overall well-being [3]. While the clinical significance of each toxicity is well documented, there remains limited understanding of their comparative mechanisms, shared predictors, and distinct risk factors—knowledge that could help guide more personalized treatment strategies.

CIN is a potentially fatal complication resulting from chemotherapy-induced hematopoietic suppression, which leads to neutropenia and heightened vulnerability to infections [1]. The US National Cancer Institute classifies CIN into four categories based on absolute neutrophil count (ANC) [4]: grade 1 (<LLN—1500/mm^3^), grade 2 (<1500—1000/mm^3^), grade 3 (<1000—500/mm^3^), and grade 4 (<500/mm^3^). Febrile neutropenia (FN), the more severe form of CIN, is defined as an ANC <1000 cells/mm^3^, accompanied by either a fever of >38.3 °C or a temperature of >38 °C lasting more than one hour [4]. FN can lead to life-threatening complications that may require urgent intervention.

In contrast, CIA impacts patients cosmetically and emotionally, as it is most often prominent on the scalp, where up to 90% of hair follicles may be in the anagen phase, making them particularly susceptible to chemotherapy-induced damage [5]. Cytotoxic chemotherapy drugs target rapidly dividing cells, leading to significant induction of apoptosis in the highly proliferative hair matrix cells [6]. The extent of CIA varies widely, depending on the specific chemotherapeutic agent, dosage, duration of therapy, route of administration, and individual patient characteristics [6]. Certain agents, such as anti-microtubular agents, have a high alopecia incidence of up to 80%, while topoisomerase inhibitors range from 60% to 100%, alkylating agents exceed 60%, and antimetabolites range from 10% to 50% [5]. Although CIA is typically reversible after chemotherapy cessation, it remains a highly distressing side effect, particularly when it affects highly visible areas such as the scalp. This visibility not only exacerbates the psychological impact on patients, but also presents a significant challenge for clinicians in managing and guiding patients through this difficult aspect of treatment.

Chemotherapy-induced neutropenia can necessitate delays or discontinuation of chemotherapy, potentially compromising patient survival outcomes. Conversely, chemotherapy-induced alopecia, while not life-threatening, significantly diminishes patients’ quality of life and may negatively influence treatment adherence. Rather than allowing toxicities to progress to a severity that necessitates treatment modification or discontinuation, it is critical to identify patients at high risk of developing these toxicities before treatment begins so that preventative interventions can be appropriately implemented to maintain treatment continuity and optimize survival outcomes. Consequently, this proactive risk stratification approach minimizes the incidence of severe adverse events and supports the delivery of effective, uninterrupted chemotherapy.

Despite the well-recognized clinical importance of both CIN and CIA, their underlying mechanisms and predictors remain insufficiently understood. This gap in knowledge is particularly pronounced for CIA, which is further complicated by the absence of FDA-approved pharmacological agents for its prevention or treatment [7]. This study seeks to bridge these gaps by providing a comprehensive comparison of CIN and CIA across key dimensions such as patient demographics, underlying malignancies, chemotherapy regimens, and genetic predispositions. By identifying shared and distinct risk factors, this research aims to empower clinicians to more effectively stratify risks, tailor treatment strategies, and ultimately enhance clinical outcomes while improving patients’ overall quality of life.

## 2. Results

### 2.1. Chemotherapy-Induced Neutropenia

#### 2.1.1. Patient-Related Risk Factors

Patient characteristics significantly influence the likelihood of developing CIN. Advanced age (>60 years) [2,8,9,10,11,12,13,14] and poor performance status [9,10,15,16,17,18], often referred to as physiological age, have emerged as critical risk factors. This reflects the reduced physiological reserve in older populations and their predisposition to experiencing adverse events.

A low body mass index (BMI) or body surface area (BSA) also predisposes individuals to more severe CIN [2,9,19,20,21,22], likely due to reduced drug clearance and altered pharmacodynamics. For example, Pettengell et al. found that, for every 10 kg increase in body weight, the risk of developing FN decreased, with an odds ratio of 0.62 [12]. Additionally, weight loss greater than 5% within the past month has been associated with a decreased risk of developing FN (*p* = 0.02) [23].

Hypoalbuminemia and malnutrition significantly increase the risk of FN (both are *p* < 0.001) [23], with odds ratios of 11.2 and 4.53, respectively [24,25]. High LDH, above the normal upper limit, and high ferritin levels, indicating inflammation, have been found to increase the risk of CIN [17,24]. An increased risk of developing CIN (grade 3 or above) has been associated with lower serum prealbumin levels and a lower prognostic nutritional index (PNI), which factors in serum albumin and lymphocyte count (both are *p* < 0.001) [26]. A meta-analysis found no significant impact of nutritional support on FN risk [27], although specific interventions such as enteral nutrition with omega-3 fatty acids, multivitamins, or vitamin E supplements were beneficial [28,29].

Additionally, various studies have observed variability in CIN risk according to sex [19,30,31,32]. While older studies suggested that females are more susceptible to developing CIN, more recent data suggest that males may experience more severe neutropenia [33].

**Figure 1:**
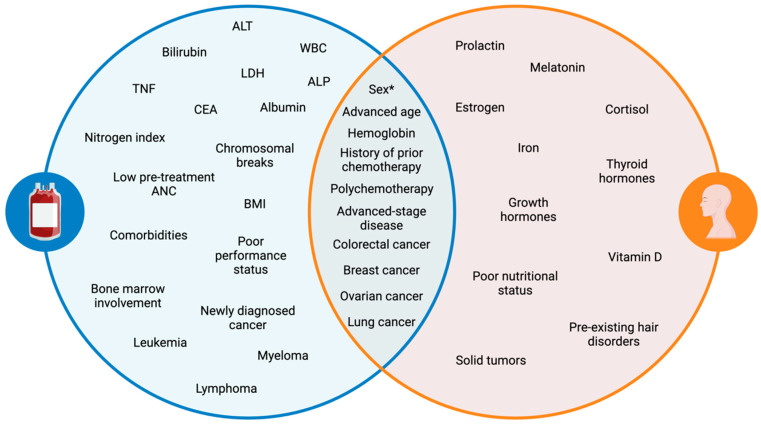
Venn diagram of patient- and disease-related risk factors for CIN and CIA. A key distinction (sex*): females are more likely to develop both toxicities; however, males may experience more severe neutropenia. Alanine aminotransferase (ALT), white blood cells (WBCs), lactate dehydrogenase (LDH), alkaline phosphatase (ALP), tumor necrosis factor (TNF), carcinoembryonic antigen (CEA).

Several laboratory anomalies are associated with an increased likelihood of developing CIN (Figure 1), including alkaline phosphatase (ALP), alanine aminotransferase (ALT), carcinoembryonic antigen (CEA), nitrogen index, serum lactate dehydrogenase (LDH), and tumor necrosis factor (TNF), along with baseline white blood cell (WBC) count, hemoglobin, bilirubin, serum albumin, and serum creatine levels [2,8,9,14,18]. A cytogenic assay can also serve as a predictor of CIN, with more than one chromosomal break per cell correlating with increased risk [8]. A low pretreatment ANC count (≤3.1 × 10^9^ L^−1^) is indicative of a higher risk of developing FN [34,35]. Moreover, lymphopenia has been linked to CIN risk, with day 1 lymphopenia (≤700 uL^−1^) following the administration of chemotherapy doubling the risk of FN development [15,36]. A follow-up study found that day 5 lymphopenia (≤700 uL^−1^) post-chemotherapy had an even stronger correlation with FN risk, showing higher sensitivity (55 vs. 8%) than the day 1 model [15]. Furthermore, baseline neutrophil count has been shown to be inversely related to the risk of CIN, with an odds ratio of 0.90 (*p* = 0.026), suggesting that higher baseline neutrophil counts reduce the likelihood of CIN [20].

The presence of comorbidities [2,37], specifically chronic obstructive pulmonary disease [32], cerebrovascular disease [32], diabetes [32], heart disease [11,32], hepatic disease [19,32], osteoarthritis [32,38], rheumatoid disease [32], and renal disease [11,38,39,40], further elevates the risk of CIN, particularly the more severe form of FN. Additionally, the total number of comorbidities, regardless of their individual subtypes, is associated with an increased risk of CIN and FN [32,37].

#### 2.1.2. Disease-Related Risk Factors

Multiple studies have demonstrated that the cancer type is a significant predictor of CIN and FN. Among all cancer-related FN hospitalizations, hematologic malignancies accounted for 59.4% of cases, followed by secondary malignancies (16.3%), breast cancer (7.8%), bone and soft tissue cancers (6.6%), and gastrointestinal cancer (5%) [41].

Patients with hematologic malignancies such as leukemia, lymphoma, and multiple myeloma are at a higher risk of developing neutropenia (Figure 1). Studies show that up to 80% of patients with hematologic cancers may develop CIN during treatment [42,43,44]. A retrospective cohort study investigating high-risk neutropenia in hematology-oncology service reported that the incidence of FN was 61.4% [44]. Among these patients, 66.6% had acute myeloid leukemia (AML) or myelodysplastic syndromes, 25.9% had non-Hodgkin’s lymphoma, 7.5% had acute lymphoid leukemia (ALL), and none had Hodgkin’s lymphoma. The increased incidence of neutropenia observed in leukemia aligns with findings from another study, which reported odds ratios of 8.87 for AML and 2.24 for ALL in predicting FN risk, further emphasizing the high susceptibility of these patients to severe neutropenia [21].

This heightened risk is in part due to the intensive chemotherapy regimens required, which cause longer-lasting neutropenia compared to those used for solid tumors [45]. AML induction therapy typically follows the 7 + 3 regimen, consisting of seven days of cytarabine combined with an anthracycline/anthracenedione for three days [46]. Anthracyclines, such as daunorubicin or epirubicin, are particularly associated with high rates of neutropenia, which may contribute to the higher incidence in AML patients [15].

Importantly, bone marrow involvement, particularly in hematological malignancies such as aggressive non-Hodgkin’s lymphoma, significantly increases the risk of severe neutropenia and FN. A study by Intragumtornchai et al. found that patients with bone marrow involvement had odds ratios of 5.8 and 4.9 for life-threatening neutropenia and FN, respectively [24]. This heightened risk stems from the structural and functional compromise of leukemic bone marrow, where the proliferation of abnormal WBCs disrupts normal hematopoiesis. Compared to healthy bone marrow, which maintains balanced hematopoiesis, leukemic bone marrow is characterized by extensive infiltration of malignant cells, leading to cytopenias such as anemia, thrombocytopenia, and leukopenia [47]. As a result, leukemic bone marrow produces fewer neutrophils at baseline and is less capable of recovery following chemotherapy, increasing the likelihood of developing neutropenia (<1000 m^3^) after treatment [48,49]. The extent of this disruption is influenced by the degree of bone marrow involvement, with acute leukemias typically exhibiting >20% marrow infiltration and a predominance of immature, nonfunctional cells, whereas chronic leukemias have <20% marrow involvement and a greater portion of mature cells. This hematopoietic fragility is reflected in the increased risk of FN seen in patients with baseline lymphopenia (OR 1.21), monocytopenia (OR 2.12), neutropenia (OR 2.84), low platelet count (OR 2.01), and anemia (OR 1.75) [21].

An estimated 10–50% of patients with solid tumors are predisposed to developing CIN [42,43]. Certain solid tumors carry a higher risk of neutropenia, with lung and breast cancer subtypes constituting the majority of neutropenia cases [50]. Additional high-risk solid tumors include colorectal and ovarian cancer. A retrospective study investigating the incidence of FN among patients with metastatic solid tumors found that metastatic lung cancer had the highest overall incidence of FN at 20.6%, followed by prostate cancer (17.7%), breast cancer (15.8%), colorectal cancer (13.7%), and ovarian cancer (13.1%) [51]. When comparing patients receiving the same chemotherapy regimen, carboplatin and paclitaxel combination therapy, the incidence of FN was higher in metastatic lung cancer patients (21.4%) compared to metastatic ovarian cancer patients (12.2%). Similar trends have been observed in prior studies, where FN occurred more frequently in advanced lung cancer patients (8%) [52] than in advanced ovarian cancer patients (4.5%) [53] following carboplatin-paclitaxel therapy.

One possible explanation for this difference is the lower utilization of prophylactic colony-stimulating factors (CSF) or antimicrobial (AMB) agents in lung cancer patients (49.1%) compared to ovarian cancer patients (57.4%) in the retrospective study [51], as prophylaxis has been shown to significantly reduce FN risk [54]. Additionally, chronic comorbidities such as cardiovascular disease and diabetes, indicating poorer baseline health, were more prevalent in lung cancer than in ovarian cancer (71.3% vs. 29.6%) [51], both of which have been associated with increased susceptibility to FN, as previously discussed. The underlying immune dysfunction and systemic inflammation associated with these conditions may have contributed to the observed higher FN rates in lung cancer patients.

The progression and severity of malignancy also strongly influence the risk of CIN. Newly diagnosed disease, advanced disease stages, and lymph node metastasis are all shown to increase the probability of developing neutropenia [24,31,55,56]. In a cohort of non–small cell lung cancer patients, severe CIN was more common in those with newly diagnosed disease (97.3%) compared to recurrent disease (2.7%) [57]. Advanced disease stage was also significantly associated with the development of severe neutropenia (*p* = 0.021), with tumor size (*p* = 0.004) and clinical stage (*p* = 0.009) being key contributing factors [58]. Although not statistically significant, lymph node metastasis also showed a notable association (*p* = 0.067).

#### 2.1.3. Treatment-Related Risk Factors

Several treatment-related factors increase the risk of CIN, including myelosuppressive chemotherapeutic regimens, duration of chemotherapy exposure, dosing schedule, and use of combination therapies.

**Figure 2:**
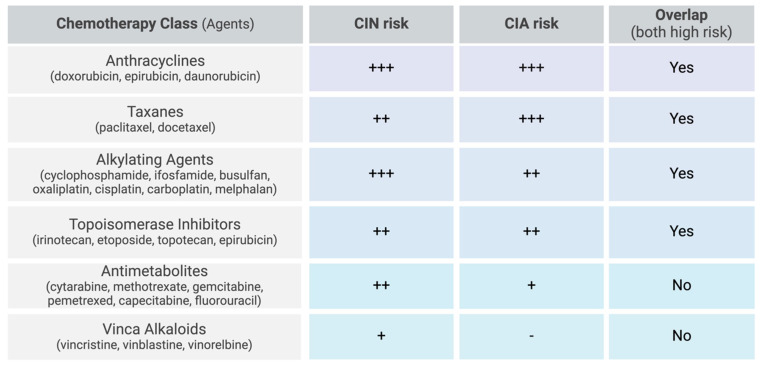
Chemotherapy-related risk factors for CIN and CIA. Scale: low (-), moderate (+), moderate-high (++), high (+++).

The most myelosuppressive chemotherapy regimens include anthracyclines, taxanes, alkylating agents, topoisomerase inhibitors, gemcitabine, and vinorelbine [2,9,33]. More specifically, high-risk chemotherapy regimens are defined as those containing doxorubicin or epirubicin ≥ 90 mg/m^2^, cisplatin ≥ 100 mg/m^2^, ifosfamide ≥ 9 g/m^2^, cyclophosphamide ≥ 1 g/m^2^, etoposide ≥ 500 mg/m^2^, or cytarabine ≥ 1 g/m^2^ per course (Figure 2) [15]. In hematological cancers, the combination of cytarabine and daunorubicin resulted in 32% of patients developing FN [44]. High-dose cytarabine alone induced FN in 24% of patients, while the combination regimen of cyclophosphamide, vincristine, doxorubicin, and high-dose methotrexate (CODOX-M) resulted in a lower incidence, with 16% of patients developing FN. For solid tumors, while prostate cancer was more strongly associated with FN overall, breast cancer patients treated with docetaxel exhibited a disproportionately high incidence of FN (21.5%) compared to prostate cancer patients receiving the same treatment (17.4%) [51].

The highest risk of neutropenia often occurs during the first cycle of chemotherapy [19,59], though a prior history of chemotherapy or polychemotherapy further increases the likelihood of developing CIN [12,33,60,61]. In a prospective observational cohort study involving 186 breast cancer patients, the incidence of grade 4 CIN was evaluated across anthracycline-based (12.9%), taxane-based (13.9%), and anthracycline-taxane combination regimens (73.2%) [62]. CIN was more frequent during the first chemotherapy cycle than the second (30.6% vs. 22.6%); however, its incidence gradually increased after multiple cycles, reaching 36.8% by the eighth cycle. This trend was partially supported by a retrospective study analyzing the incidence of FN in advanced breast cancer patients receiving adjuvant adriamycin and cyclophosphamide followed by docetaxel (TAC regimen), where 54.5% of patients experienced FN overall. Specifically examining individual chemotherapy cycles, FN incidence was highest during the first cycle (34.2%) and decreased to 18.2% in the second cycle, ultimately declining to 15% after six cycles [63]. Neoadjuvant treatments typically cause higher incidence and severity of CIN than palliative treatments due to their aggressive regimen intensity. For instance, a study by Phua et al. evaluating metastatic breast cancer patients receiving palliative therapy with the FEC regimen (5-FU, epirubicin, and cyclophosphamide) reported notably lower rates of FN (6.2%), highlighting the relationship between reduced treatment intensity and decreased neutropenia risk [64].

Regimens administered at less frequent intervals were more likely to cause CIN compared to those with more frequent dosing. A study in ovarian cancer patients evaluated the efficacy and toxicity profiles of conventional combination therapy of paclitaxel and carboplatin (paclitaxel: 175 mg/m^2^ over 3 h, carboplatin: AUC 6; q3W every 6 cycles) to a dose-dense regimen (paclitaxel: 80 mg/m^2^ over 1 h, carboplatin: AUC2; weekly for 18 cycles) [65]. Dose-dense regimens of paclitaxel resulted in fewer adverse events, with grade 3–4 neutropenia occurring in 42% of patients rather than 50% in the conventional regimen (*p* = 0.021) and FN occurring in 0.5% of cases compared to 3% (*p* = 0.012).

Combining chemotherapy also increases the likelihood of developing FN. Breast cancer patients treated with taxane combinations had the highest incidence of CIN and FN, with the docetaxel and cyclophosphamide regimen resulting in 20.9% of patients developing FN and 53.6% having severe neutropenia [66]. The combination of docetaxel with carboplatin and trastuzumab raised FN incidence to 25.1%, with 62.8% of patients developing severe neutropenia. Other combination regimens also showed notable rates of severe neutropenia, such as in colorectal cancer patients, where the capecitabine and oxaliplatin combination (XELOX) caused 27.2% of patients to develop severe neutropenia, and the leucovorin, 5-fluorouracil, and oxaliplatin combination (FOLFOX6) resulted in 47% of patients developing grade 3–4 neutropenia.

#### 2.1.4. Genetic Risk Factors

**Figure 3:**
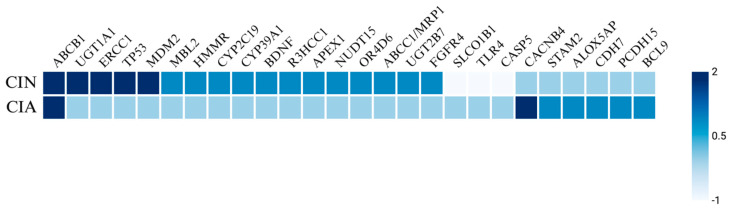
Heatmap of genetic predisposing factors for CIN and CIA. The heatmap illustrates the genetic factors influencing chemotherapy-induced neutropenia (CIN) and chemotherapy-induced alopecia (CIA). Scoring key: 2 indicates a highly significant increase in incidence or severity, 1 suggests a potential increase in incidence, 0 denotes no association, and −1 reflects a decreased incidence.

Genetic factors including specific genotypes and SNPs have been identified as risk factors for the development of CIN and FN in various cancers. Notable SNPs associated with elevated incidence and severity of neutropenia include those in the ABCB1 [35,67,68,69,70], UGT1A1 [71,72,73], ERCC1 [35,69], TP53 [74,75], MDM2 [74,75], and SLCO1B1 [73,76] genes (Figure 3). A retrospective cohort study of 105 Taiwanese breast cancer patients treated with palbociclib, a targeted CDK4/6 inhibitor, examined the prevalence of neutropenia associated with four SNPs: ABCB1_rs1045642, ABCB1_rs1128503, ERCC1_rs3212986, and ERCC1_rs11615 [69]. The study found that 70.4% of patients developed severe neutropenia, with a higher SNP frequency of the T allele in ABCB1_rs1128503 and the G allele in ERCC1_rs11615. Similarly, a replication study involving 108 solid tumor patients treated with irinotecan analyzed the ANC nadir for UGT1A1 and SCLO1B1 variants [73]. This study reported that UGT1A1*28 and UGT1A1*93 both yielded a decreased ANC nadir, while SLCO1B1*1b was associated with an increased ANC nadir. In a study by Innocenti et al., the same variants of UGT1A1 and SLCO1B1 were linked to a decreased ANC nadir, alongside the ABCC1 IVS11-48C>T variant [77]. Okishiro et al. studied breast cancer patients treated with the combination regimen of 5-fluorouracil, epirubicin, and cyclophosphamide (FEC) and found that genetic polymorphisms in MDM2 SNP309 and TP53 R72P were associated with severe neutropenia and FN, particularly with the C/C genotype in TP53 and the T/T + T/G genotype in MDM2, which correlated with an 83.3% incidence of severe neutropenia and a 62.5% incidence of FN [75].

Additionally, polymorphisms have been identified in isolated studies, including SNPs in HMMR [78], CYP2C19*2 [79], CYP39A1 [80], BDNF [81], R3HCC1 [82], APEX1 [83], NUDT15 [83], OR4D6 [84], ABCC1/MRP1 [85], UGT2B7 [85], FGFR4 [85], and MBL2 genes [86]. Kim et al. investigated 12 gene variants in 185 pediatric ALL patients treated with mercaptopurine and reported that APEX1_rs2307486 variants (G/G genotype) were significantly associated with the highest cumulative incidence of neutropenia, while NUDT15_rs116855232 variants (T/T genotype) were strongly linked to a higher cumulative incidence of neutropenia and reduced chemotherapy tolerance [83]. Moreover, in a large retrospective study of 1012 breast cancer patients treated with FEC, variants in ABCC1/MRP1_rs4148350, ABCC1/MRP1_rs246221, and ABCC1/MRP1_45511401 showed a statistically significant correlation with FN, although only the first two were also associated with prolonged neutropenia [85]. Furthermore, UGT2B7_rs76688282 was correlated with both FN and prolonged severe neutropenia, while FGFR4_rs351855 was linked to the development of FN. Mixed findings were reported for TGFB1_rs1800469, where one copy of the wild-type allele reduced the probability of CIN, while two copies increased the likelihood of developing CIN [81]. Some polymorphisms, including TLR4 and CASP5, have been associated with a decreased probability of developing neutropenia [81].

### 2.2. Chemotherapy-Induced Alopecia

#### 2.2.1. Patient-Related Risk Factors

Patient-related characteristics are crucial for predicting the risk of hair loss in patients undergoing chemotherapy. Hair loss consistently ranks among the most distressing side effect of chemotherapy, making it essential to set realistic expectations pre-treatment for patients based on their risk factors. Key patient-related risk factors include sex, age, comorbid hair disorders, pretreatment hair characteristics, hormonal status, and nutritional status [87,88].

Abdel-Rahman found that, among patients with metastatic colorectal cancer receiving FOLFOX-based regimens, 20% of women experienced alopecia compared to 8.6% of men [89]. Similarly, Can et al. reported a higher incidence of complete alopecia in women (52.5%) compared to men (13.9%) across patients with various cancers, including lung, breast, urologic, and hematologic malignancies [90].

Age is another important factor influencing the risk and severity of CIA. Older patients may be more susceptible to developing CIA and may experience more severe hair loss [5,88,91], likely due to increased sensitivity of hair follicles to chemotherapy drugs and a slower hair growth cycle, which can affect both hair loss and regrowth. However, one study in breast cancer patients found no association between age and the incidence or severity of hair loss; instead, age appeared to aid in the recovery from CIA, with younger premenopausal women experienced significantly faster regrowth of eyebrows, eyelashes, and body hair than older postmenopausal women [92]. Additionally, older patients were more likely to experience thinner hair diameters after chemotherapy than their younger counterparts.

The presence of pre-chemotherapy hair disorders, such as androgenetic alopecia, may be a predisposing factor for the development of CIA [88,91]. Bergfield et al. reported that damaged, tinted, permed, or bleached hair may increase the risk of alopecia [93]. Diagnostic tools such as the pull test, trichogram, and trichoscopy can provide valuable insights into the hair cycle and reveal underlying abnormalities that could increase the susceptibility to further hair damage by chemotherapy. For example, patients with fewer hairs in the anagen phase at the time of induction will be less sensitive to the effects of chemotherapy.

Blood levels of hemoglobin, iron, thyroid hormones, and vitamin D may help further stratify the risk of CIA [94,95,96]. The active form of vitamin D has been described as a potential therapy for CIA [97], as it may promote functional differentiation of dermal papilla cells [98]. Hormones, such as estrogen, prolactin, thyroid hormone, cortisol, growth hormone, and melatonin, have also been implicated in hair growth regulation [96].

Finally, nutritional status plays an important role in the development of chemotherapy toxicity, including CIA. Arrieta et al. found that, among non–small cell lung cancer patients receiving cisplatin plus paclitaxel, 84% developing CIA after two cycles [91]. Among these patients, those who were moderately or severely malnourished with hypoalbuminemia were more likely to experience increased chemotherapy-induced toxicity compared to well-nourished patients (31% vs. 22%; *p* = 0.02) and those with normal albumin levels (54% vs. 41%; *p* = 0.04). Here, researchers evaluated malnutrition by assessing factors including weight loss (past 6 months), fluid balance, subcutaneous fat, and muscle mass. Buyukavci et al. observed that hair contents of iron and zinc did not impact the development of CIA [99], while Sieja et al. demonstrated that selenium ingestion significantly decreased the risk of developing CIA [100]. In a phase I study, topical calcitriol (1,25-dihydroxyvitamin D_3_) reduced CIA [101]. Given this evidence and the association between vitamin D deficiency and various forms of alopecia, it is plausible that maintaining adequate levels of vitamin D may help mitigate the risk or severity of CIA [102].

#### 2.2.2. Disease-Related Risk Factors

Certain disease-related factors influence the risk and severity of CIA, primarily based on cancer type and stage. Breast cancer is among the most studied malignancies regarding CIA, given the frequent use of anthracycline- and taxane-based regimens, such as paclitaxel and docetaxel, which are strongly associated with alopecia [103]. A prospective cohort study of breast cancer patients found that 11.5% experienced permanent CIA (pCIA) six months following chemotherapy, with the combination of doxorubicin and cyclophosphamide increasing the risk [104]. A review of long-term outcomes reported that 46.1% of breast cancer patients still exhibited pCIA three years after treatment [105].

Although CIA research primarily focuses on breast cancer, it also occurs in other solid tumors, though its incidence varies widely depending on treatment regimens. For example, an analysis of CIA following docetaxel therapy revealed lower incidence in metastatic prostate cancer (34.3%) and non–small cell lung cancer (37.7%) compared to recurrent breast cancer (83.3%) [106]. Freites-Martinez et al. reported that CIA incidence approached 100% for docetaxel across several cancers, including breast, gastric, head and neck, lung, and prostate [107]. Similarly, paclitaxel showed near 100% CIA rates in breast, lung, and gynecologic malignancies, as well as Kaposi’s sarcoma [107]. A review by Saraswat et al. also highlighted the high incidence of CIA in rectosigmoid cancer (22.9%) and lung cancer (25.6%) [108]. A phase I and preliminary phase II study documented a 90% incidence of alopecia following adriamycin treatment across multiple malignancies, including breast, lung, thyroid, and hematologic cancers [109]. In this study, hematologic cancers included ALL, acute lymphosarcoma cell leukemia, acute erythroleukemia, chronic myelogenous leukemia (CML), and chronic lymphocytic leukemia (CLL).

In contrast, hematologic malignancies have received less attention in CIA research, although studies indicate significant variation in alopecia incidence depending on the chemotherapy regimen. For instance, there was an 11% incidence of alopecia in AML patients receiving pirarubicin and cytarabine [110], compared to 100% in acute non-lymphocytic leukemia patients treated with idarubicin and cytarabine [111]. Freites-Martinez et al. reported that CIA rates in hematologic cancers ranged from 50% to 100% depending on the drug used [107]. Doxorubicin was associated with an incidence greater than 80% in AML, ALL, and lymphoma. Idarubicin-induced CIA was estimated at approximately 50% in AML, whereas daunorubicin was linked to 100% CIA in both AML and ALL.

Interestingly, CIA in hematologic malignancies may be linked to improved treatment response. A scoping review on Hodgkin’s lymphoma found that, among the 52% of patients who experienced complete alopecia, those with more severe hair loss had a higher likelihood of achieving remission [112]. A retrospective analysis further supported this association, showing that 83% of Hodgkin’s patients who achieved complete remission also developed total hair loss [113]. These findings suggest that CIA severity may serve as an indirect marker of chemotherapy regimen efficacy in certain hematologic malignancies.

#### 2.2.3. Treatment-Related Risk Factors

The severity of CIA is influenced by several factors, including the specific chemotherapy agent, dose, administration schedule, and route of delivery. The mechanism of action plays a critical role in predicting CIA risk.

Certain chemotherapy agents are more strongly associated with CIA than others. Taxanes, anthracyclines, topoisomerase inhibitors, and alkylating agents have the highest incidence, with some regimens resulting in alopecia in nearly 100% of treated patients [5]. Specifically, the incidence of alopecia is 60–100% with topoisomerase inhibitors (topotecan, etoposide, irotecan, and epirubicin), 80% with anti-microtubular agents (paclitaxel and docetaxel), over 60% with alkylating agents (cisplatin, carboplatin, cyclophosphamide, ifosfamide, and melphalan), and 10–50% with antimetabolites (gemcitabine and pemetrexed) (Figure 2) [5].

A scoping review of CIA in ovarian cancer reported that alopecia incidence ranged from as low as 2% (all grades) in patients receiving cisplatin monotherapy to 100% (grade 1–2) in those treated with docetaxel monotherapy [114]. Similarly, a review found that CIA incidence in lung cancer patients ranged from 49% with topotecan to nearly 100% with docetaxel, doxorubicin, or paclitaxel [107]. In contrast, irinotecan was associated with a lower CIA incidence of approximately 58% in colorectal cancer, while etoposide-induced alopecia occurred in 55% of patients with small cell lung cancer or testicular cancer [107]. A review on CIA in ovarian cancer patients reported that taxane monotherapy had the highest alopecia incidence, with docetaxel leading to alopecia in 100% of patients and paclitaxel in 62.8–79% [114]. In contrast, treatment with 5-fluorouracil, methotrexate, vinca alkaloids (vinorelbine and vinblastine), erlotinib, gefitinib, or pembrolizumab had a lower incidence and severity of alopecia [87,93,107,115,116,117,118]. A phase III trial of vinorelbine in stage III–IV non–small cell lung cancer found that only 11.4% of patients developed grade 1 alopecia, and 8.9% experienced grade 2 alopecia [119].

Combination therapy is frequently used for aggressive malignancies but often results in a higher incidence of CIA due to cumulative cytotoxic effects. The ICON4 trial illustrated this effect, reporting an alopecia incidence of 86% for patients receiving paclitaxel combined with platinum-based (PB) chemotherapy, compared to only 25% with PB monotherapy [86]. Polychemotherapy, which is commonly used in breast cancer treatment, can further exacerbate hair loss. For instance, docetaxel combined with cyclophosphamide resulted in a CIA prevalence of 96.7–100% compared to 83.3% for docetaxel monotherapy [106].

Liposomal drug delivery has emerged as a potential strategy to reduce CIA by minimizing off-target toxicity [120]. In metastatic breast cancer, liposomal doxorubicin resulted in a significantly lower incidence of alopecia (20%) compared to conventional doxorubicin (66%) [120]. Similarly, in soft tissue sarcoma patients, pegylated liposomal doxorubicin reduced CIA incidence to 6% compared to 86% with standard doxorubicin [121]. In breast cancer patients, the combination of docetaxel and cyclophosphamide with liposomal doxorubicin reduced CIA incidence from 96.7–100% to 64.3% with standard doxorubicin [106].

Both the cumulative dose and the administration schedule play critical roles in determining CIA severity. Higher cumulative doses and shorter infusion intervals increase hair follicle toxicity, particularly during the anagen phase, often correlating with more severe alopecia [122,123]. Low-dose cyclophosphamide treatment induced alopecia in 25% of patients, while high-dose cyclophosphamide resulted in 100% incidence among patients with leukemia, lymphoma, multiple myeloma, breast cancer, neuroblastoma, retinoblastoma, or ovarian cancer [107]. Dose schedules significantly impact CIA incidence in ovarian cancer patients [114]. Weekly paclitaxel administration resulted in 46% CIA compared to 79% with every-three-week dosing. Similarly, weekly carboplatin-paclitaxel treatment led to 29% incidence of CIA versus 59% with an every-three-week schedule.

Although CIA is typically reversible, certain agents such as busulfan and taxanes pose a higher risk of pCIA [124,125,126,127,128], likely because of irreversible damage to hair follicle stem cells [88]. pCIA is defined by the absence of scalp and body hair regrowth six months after the discontinuation of chemotherapy [126]. A retrospective study by Palamaras et al. reported pCIA incidences ranging from 0.8% to 71% in adults and 24% in children treated with busulfan, with or without cyclophosphamide [127]. Chan et al. specifically highlighted variations in pCIA risk among different taxanes [129]. In breast cancer patients, those receiving docetaxel had a significantly higher incidence of pCIA (23.3%) compared to those treated with paclitaxel (10.1%).

#### 2.2.4. Genetic Risk Factors

Genetic variations may influence the susceptibility to CIA. SNPs near genes such as CACNB4, STAM2, and ABCB1 are associated with a higher risk of CIA, particularly in breast cancer cohorts. Chung et al. reported several SNPs near genes encoding CACNB4, PCDH15, STAM2, ALOX5AP, BCL9, and CDH7 associated with increased risk of CIA in a cohort of Japanese patients with breast cancer who were treated with chemotherapy [103]. Specifically, CACNB4 was significantly associated with drug-induced grade 2 alopecia, whereas the other SNPs were suggestively associated with CIA (Figure 3). Similarly, Núñez-Torres et al. conducted a genome-wide association study on women with breast cancer undergoing chemotherapy and found a significant association between a regulatory variant of the ABCB1 gene and the occurrence of persistent CIA [130]. The ABCB1 gene is expressed in human hair follicle stem cells and encodes an efflux pump named P-glycoprotein that is responsible for transporting drugs, including docetaxel, out of cells. Carriers of a specific risk allele identified in this study exhibited decreased ABCB1 expression, which likely leads to lower levels of P-glycoprotein and, consequently, reduced efflux of docetaxel. The intracellular accumulation of the toxic drug may result in permanent destruction of the hair follicle stem cells and therefore pCIA.

## 3. Discussion

This comparative analysis summarized various risk factors associated with CIN and CIA, both of which stem from the cytotoxic effects of chemotherapy on proliferative cells, namely hematopoietic stem cells and proliferative hair follicle cells. These risk factors were categorized into patient-related, disease-related, treatment-related, and genetic factors.

Physiological aging emerged as a significant risk factor for both CIN and CIA, likely due to reduced cellular regenerative capacity, with older patients (>60 years) being at a higher risk for both adverse events. While older patients showed delayed hair regrowth in CIA, aging does not have a similar effect on recovery from CIN, which is more dependent on the severity and duration of neutropenia. Comorbidities such as diabetes, cardiovascular disease, and renal impairment exacerbate CIN severity, while pre-existing hair disorders such as androgenetic alopecia, as well as poor nutritional status, contribute more significantly to CIA risk and severity. For instance, low BMI or low BSA is linked to an increased CIN risk, likely due to altered drug pharmacokinetics, while iron and vitamin deficiencies contribute to an increased CIA risk. Sex differences also show contrasting patterns for these toxicities: while females tend to experience higher rates of CIN and CIA, males may experience more severe CIN.

Laboratory markers play a crucial role in predicting both CIN and CIA risk. For CIN, abnormalities in WBC count, ANC, lymphocyte levels, and inflammatory markers such as LDH and TNF have been linked to increased susceptibility. Low ANC and lymphopenia, in particular, serve as strong predictors of FN. On the other hand, for CIA, deficiencies in hemoglobin, iron, thyroid hormones, and vitamin D have been associated with more severe hair loss. Whereas CIN primarily reflects the body’s ability to respond to infections and immune system stress, CIA underscores the impact of systemic health, particularly nutrition and hormonal balance, on the health of hair follicles.

Genetic predisposition also plays a role in the development of these complications; however, no clear overlap has been identified between the two adverse effects of chemotherapy in the current literature besides polymorphisms in the ABCB1 gene (Figure 3). SNPs in genes such as ABCB1, UGT1A1, ERCC1, TP53, MDM2, BDNF, APEX1, NUDT15, and MBL2 were associated with a higher CIN risk, whereas SNPs in genes such as CACNB4, STAM2, ABCB1, PCDH15, ALOX5AP, BCL9, and CDH7 have been associated with a higher CIA risk.

Cancer type is another significant disease-related factor influencing the risk of CIN and CIA. Hematologic malignancies such as leukemia, lymphoma, and multiple myeloma carry a high risk for CIN, with up to 80% of patients experiencing neutropenia during treatment. Specifically, FN incidence is highest in AML and myelodysplastic syndromes, where patients also experience longer durations of neutropenia and increased susceptibility to infection. This heightened risk is linked to the fact that hematological malignancies directly affect the bone marrow, where hematopoiesis occurs, making these patients more susceptible to neutropenia during chemotherapy [45]. Conversely, CIA is less frequently studied in hematologic malignancies, but chemotherapy agents such as doxorubicin and daunorubicin have been associated with high alopecia rates in patients with these cancers. For instance, studies of AML and ALL report CIA incidences of up to 100% with those drugs [107]. Both CIN and CIA in hematologic cancers have been linked to better treatment outcomes, suggesting a potential prognostic role for these toxicities. Severe CIN has been associated with improved overall survival and increased remission likelihood, indicating that neutropenia may serve as a marker of effective chemotherapy response [131].

In contrast, solid tumors generally pose a lower CIN risk compared to hematologic cancers. However, lung, breast, colorectal, and ovarian cancers remain significant contributors to both CIN and CIA risk. For CIN, metastatic disease further elevates the likelihood of FN, with lung cancer patients exhibiting a 20.6–21.4% FN incidence. Interestingly, ovarian cancer patients receiving the same chemotherapy regimen experienced lower rates of CIN (13.1%), suggesting that, even with similar treatments, solid tumors may present a slightly lower but still significant risk for neutropenia. For CIA, solid tumors, particularly breast cancer, have been the focus of most research. Breast cancer patients, especially those treated with taxane- and anthracycline-based regimens, experience high rates of CIA, with around 46.1% exhibiting pCIA three years after chemotherapy treatment. This is in contrast with the incidence of alopecia in other solid tumor cases, such as ovarian and lung cancers.

Both CIN and CIA in solid tumor cases are strongly influenced by disease progression and staging. Newly diagnosed cancers are more strongly associated with severe CIN compared to recurrent disease, with advanced-stage malignancies further increasing susceptibility. Tumor size, clinical stage, and lymph node metastasis all play significant roles in determining CIN severity. Similarly, bone marrow involvement significantly lowers neutrophil counts both at baseline and post-treatment, further predisposing patients to severe neutropenia. As for CIA, patients with advanced cancers, such as breast, ovarian, and lung cancers, tend to experience higher rates of hair loss, with specific regimens like docetaxel inducing alopecia in nearly 100% of treated patients.

Patients are at the highest risk of FN during the first cycle of chemotherapy, with most neutropenic events occurring between days 10 and 14 after chemotherapy initiation [132]. In contrast, Watanabe et al. observed that the mean time from chemotherapy induction to hair loss onset was approximately 18 days, with near-complete hair loss occurring within 2–3 months [133]. Both cytotoxic events occur during treatment course, and the resulting delays or reductions in chemotherapy regimens due to CIN and FN can have significant financial and long-term survival implications. These toxicities require close monitoring and comprehensive patient education in order to improve patient outcomes while minimizing healthcare costs.

Given that CIN and subsequent FN are major dose-limiting toxicities in patients receiving chemotherapy, the most effective form of management is prevention, particularly with the use of granulocyte colony-stimulating factor (G-CSF) [33]. Risk assessment models have been developed to predict CIN in high-risk groups to optimize treatment adherence without dose reductions or delays [20,134]. These models aim to guide the targeted use of G-CSF, thereby increasing the proportion of patients who can complete their full chemotherapy course without major complications. This approach has the potential to improve treatment outcomes while reducing healthcare costs [135]. Current guidelines from the American Society of Clinical Oncology (ASCO) recommend the use of prophylactic G-CSF if the risk of FN is ≥20% [136].

In contrast, the management of CIA remains largely supportive, despite hair loss being consistently cited as one of the most traumatic aspects of chemotherapy. Currently, there are no pharmacological treatments to prevent chemotherapy-induced hair loss. Aside from various scalp cooling devices that have limited use and efficacy, there is no FDA-approved treatment for CIA. However, interventions such as platelet-rich plasma and minoxidil are being actively tested [102], and liposomal drug delivery may offer a promising future strategy to reduce CIA incidence once it becomes available. The current lack of effective treatments underscores the critical role that clinicians must play in providing supportive care to help mitigate patient distress, improve adherence to chemotherapy, and address psychological well-being.

This study had several limitations. First, while we identified risk factors for both CIN and CIA, the existing literature on genetic risk factors is limited. Moreover, no studies have yet compared these genetic factors across CIN and CIA, and further research is needed to clarify potential genetic predispositions. Additionally, although certain chemotherapeutic agents are commonly associated with CIN and CIA, variability in treatment regimens, patient populations, and dosages complicates the generalization of these findings across different clinical settings. Lastly, this analysis primarily focused on the clinical and physiological aspects of CIN and CIA and did not fully address the psychological and quality-of-life impacts of these adverse reactions, which remain a significant concern for patients undergoing chemotherapy.

Our findings warrant further investigation to better understand the relationship between CIN and CIA. Increasing efforts in comprehensive genomic studies may help identify genetic risk factors for both adverse reactions, enabling clinicians to optimize treatment protocols. In addition, our study sheds light on the urgent need for research into novel pharmacological treatments for CIA, as the current options are limited and often ineffective. Despite these limitations, this review serves as the first direct comparison between CIN and CIA, providing valuable insights that can inform personalized oncology care.

## 4. Conclusions

By delineating shared and unique predictors of CIN and CIA, this study provides a foundation for integrated toxicity management. Although both conditions result from the impact of chemotherapy on rapidly dividing cells, their distinct timelines, clinical effects, and patient burden highlight the need for tailored interventions. Future studies should explore mechanistic overlaps and novel therapeutic approaches to mitigate these toxicities, particularly in high-risk populations.

## Abbreviations

CIN	Chemotherapy-induced neutropenia
CIA	Chemotherapy-induced alopecia
FN	Febrile neutropenia
